# Responding to adverse patient safety events in Viet Nam

**DOI:** 10.1186/s12913-019-4518-y

**Published:** 2019-09-18

**Authors:** Reema Harrison, Anurag Sharma, Merrilyn Walton, Esmond Esguerra, Seinyenede Onobrakpor, Bui Trung Nghia, Nguyen Duc Chinh

**Affiliations:** 1 Faculty of Medicine, School of Public Health and Community Medicine, UNSW Sydney, Room 308, Samuels Building (F25), Sydney, NSW 2052 Australia; 2 Faculty of Medicine and Health, School of Public Health, The University of Sydney, Sydney, Australia; 3 Office for Global Health Faculty of Medicine and Health, The University of Sydney, Sydney, Australia; 4 Viet Duc University Hospital, Hanoi, Vietnam

**Keywords:** Adverse events, Incident reporting, Health policy, Hospitals, Patient safety, Survey research

## Abstract

**Background:**

The psychological and professional impact of adverse events on doctors and nurses is well-established, but limited data has emerged from low- and middle-income. This article reports the experiences of being involved in a patient safety event, incident reporting and organisational support available to assist health professionals in Viet Nam to learn and recover.

**Method:**

Doctors and nurses (1000) from all departments of a 1500-bed surgical and trauma hospital in Viet Nam were invited to take part in a cross-sectional survey. The survey explored respondents’ involvement in adverse events and/or near miss, their emotional, behavioural and coping responses, experiences of organisational incident reporting, and the learning and/or other consequences of the event. Survey items also assessed the availability of organisational support including peer support and mentorship.

**Results:**

Of the 497 respondents, 295 (59%) experienced an adverse event in which a patient was harmed, of which 86 (17%) resulted in serious patient harm. 397 (80%) of respondents experienced a near miss, with 140 of these (28%) having potential for serious harm. 386 (77%) reporting they had been affected professionally or personally in some way, with impacts to psychological health (416; 84%), physical health (388; 78%), job satisfaction (378; 76%) and confidence in their ability (276; 56%) commonly reported. Many respondents were unable to identify local improvements (373; 75%) or organisation-wide improvements following safety events (359; 72%) and 171 (34%) admitted that they had not reported an event to their organisation or manager that they should have.

**Conclusions:**

Health professionals in Viet Nam report impacts to psychological and physical health as a result of involvement in safety events that reflect those of health professionals internationally. Reports of limited organisational learning and improvement following safety events suggest that patient safety culture is underdeveloped in Viet Nam currently. In order to progress work on patient safety cultures and incident reporting in Viet Nam, health professionals will need to be convinced not only that they will not be exposed to punitive action, but that learning and positive changes will occur as a result of reporting safety events.

## Background

An adverse event in healthcare is described as *“an injury related to medical management, in contrast to complications of disease*) [1]. Around 10% of patients in developed countries will experience preventable harm during their course of the medical care, with higher estimates obtained from low- and middle-income settings [2, 3]. Over the past 30 years, many governments and health systems have invested in resources to reduce preventable harm to patients, with incident reporting and retrospective analysis of adverse and near-miss events being a core component of this work [4, 5]. Health systems that measure and report incidents serve an important role in raising awareness of the potential for errors as well as promoting safety cultures [6]. Incident reporting is not a tool that directly remedies problems arising in care; rather it provides a surveillance process that enables exploration and assessment of the risks posed to patients and staff. Reporting, therefore continues to be a foundational process underpinning the development of a patient safety culture. Retrospective analysis of system vulnerabilities is a valuable method, with incident reporting policies and tools embedded in many healthcare system, particularly in high income countries [5, 7, 8]. Reporting plays a role in promoting resilient systems, alongside proactive attempts to optimise the provision of care [9].

Patient safety research has gained momentum over the past 30 years, building knowledge of the key patient safety concerns arising in many developed countries. Less is known about patient safety in low-resource settings. Some low- and middle-income countries are proactively incorporating patient safety goals into national policies, although many countries have yet to collect incident reports at a national level [10–12]. In 2013, the Ministry of Health in Viet Nam mandated a national policy of incident reporting for hospitals. However, since the policy directive there is little evidence of incident reporting systems in hospitals, or where they exist, of being utilised to facilitate learning.

A key feature of organisations using incident reporting is that their health professionals are supported by their organisation; that they feel free to discuss and learn from errors and that a just culture exists [13]. Evidence to date shows that clinicians who directly or indirectly contribute to an adverse event can experience psychological effects that disrupt their professional and personal lives and their ability to deliver high quality, safe care [14–18]. Anxiety, depression, sleep disturbance, fear and worry are consistently reported as well as shame, guilt, loss of self-confidence and feelings of incompetence and worthlessness [19–22]. The severity of these effects is often related to the degree of harm to the patient; they are more pronounced with more serious incidents [19, 22].

The detrimental effects following an incident can further harm patients, clinicians, and the wider healthcare system; they can also have flow on effects such as a reduced likelihood of open and honest reporting and discussion of mistakes and adverse events [23]. Safety-conscious industries such as aviation recognize that front line staff will only speak up if they feel supported, have confidence that they will be treated fairly, and that their reports will be used for learning rather than punishment [13]. Until this study, the experiences of health professionals in Viet Nam regarding their experiences of adverse events, of incident reporting or of the organisational support available for health professionals involved in safety events to learn and recover has been unknown. This study therefore aimed to contribute to understanding of these phenomena, and to also explore wider implications for the development of safety culture in Viet Nam.

## Methods

### Sample and setting

Doctors and nurses from all departments of a 1500-bed surgical and trauma and teaching hospital in Viet Nam were invited to participate in the study, with 1000 potential participants invited in total. The study site is a leading surgical hospital for the treatment of injuries, with most patients experiencing trauma in rural and urban regions transferred to the hospital for treatment. The study site operates a system of incident report from doctors, nurses and other staff via the Nursing Department. Anonymous daily and monthly reports are processed through the Quality Control Department. The Quality Control Department staff, with the assistance of the General Planning Department, perform analyses to identify actual or potential medical errors then undertake root -cause analyses of these events. All the reports and analysis are sent to the Directorial Board or presented at weekly meetings in the hospital with the Directors and representatives of all departments present in order to identify solutions and develop interventions where required.

### Survey tool

This survey tool used is a validated instrument previously used with United Kingdom (UK), United States of America (US) and Australasian health professionals, but adapted and translated for use with a Vietnamese sample [24, 25]. Standard definitions were used to explain the terms ‘adverse events’ and ‘near misses’. Data were collected regarding respondents’ involvement in adverse events and/or near miss, their emotional, behavioural and coping responses, experiences of organisational incident reporting, and the learning and/or other consequences of the event. Survey items also assessed the availability of organisational support including mentorship. The translated survey was independently checked by the Vietnamese bilingual research team members to ensure accuracy.

### Procedure

The paper-based survey was administered to doctors and nurses across all departments of an urban surgical hospital in Viet Nam. The Vietnamese research assistant delivered survey packs containing an invitation letter and the survey to doctors and nurses in each department of the hospital and then collected the anonymous, completed surveys one week later. No identifiable information was collected.

### Analysis

**Fig. 1:**
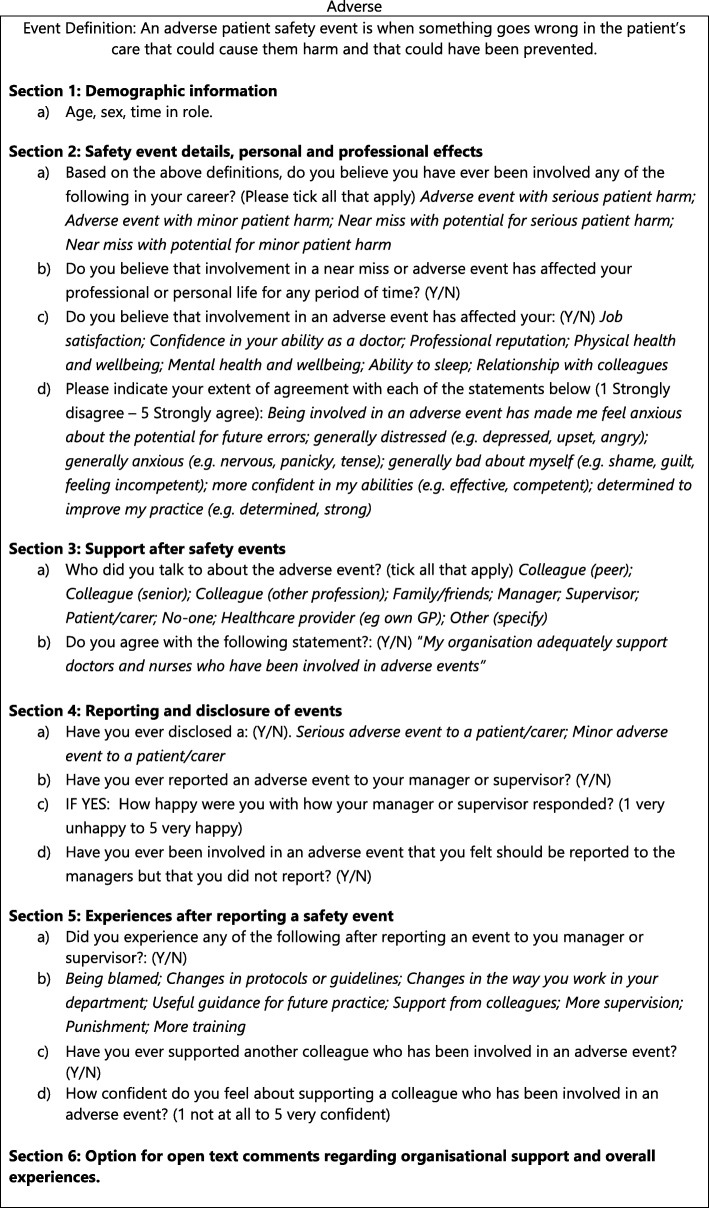
Summary of survey components

The outcome variables of interest were derived from responses to the questionnaire items including involvement in an incident and a range of associated personal and professional outcomes (Fig. 1). Involvement in a near miss or adverse event was captured through four response options: i) adverse event with serious patient harm, ii) adverse event with minor patient harm, iii) near miss with potential for serious patient harm, iv) near miss with potential for minor patient harm or v) none of these. Descriptive statistical analysis was undertaken using the Stata 15 package.

## Results

Of the 1000 invited participants, 497 responded (50% response rate). Table 1 displays the demographic data relating to the sample. Most respondents were aged 25–44 years (403; 81%) and mid-career (273; 55%), with a slightly stronger representation of female respondents (300; 60%). A cross-section of specialty backgrounds was apparent, with anesthesia (119; 24%), orthopedics (69; 14%) and emergency care (58; 12%) as the largest specialty groups, reflecting the status of this hospital as a trauma and surgical centre.

**Table 1:** Descriptive Statistics: Demographics

Covariates	N	%
Experience (Years in Practice)		
< 2 Years	84	16.99
2–10 Years	273	54.96
> 10 Years	140	28.17
Age Categories (%)		
< 25 years	55	11.07
25–34 years	296	59.56
35–44 years	107	21.53
45–54 years	32	6.44
55–64 years	7	1.41
Gender (%)		
Male	197	39.64
Female	300	60.36
Speciality (%)		
Anaesthesia	119	23.94
Orthopaedic	69	13.88
ED	58	11.67
Spinal	32	6.43
Surgery	32	6.44
Other	187	37.64

Number of Observations (N): 497

Table 2 outlines the nature of adverse events and near miss events experienced by the respondents. Just over half experienced an event harming a patient, 295 (59%) of which 86 (17%) experienced an event resulting in serious patient harm. Most respondents reported experiencing a near miss (397; 80%), with 140 of these (28%) having potential for serious harm. Many respondents had disclosed their adverse event (172; 35%) or near miss (233; 47%) to a patient or carer. Most respondents had reported an event to a manager (407; 82%), but 171 (34%) admitted that there had been events that they had not reported to their organisation or manager when they should have.

**Table 2:** Descriptive Statistics: Outcome Variables

Incident type	N
Serious Harm	
Actual	86
Near miss	140
Minor Harm	
Actual	209
Near miss	257
Incident Reporting	
Disclosed minor event to patient/carer	233
Disclosed serious event to patient/carer	172
Reported near miss/serious event to manager	407
Not reported an event that you should have	171

Number of Observations (N): 497

Table 3 shows little learning and change occurred after reporting an incident. All respondents reported ‘slightly’ or ‘not at all’ when answering this question despite the five-point response scale provided. Most respondents said they received little useful feedback or empathy from their colleagues (426; 86%), with many unable to identify local improvements (373; 75%) or organisation-wide improvements such as changes to protocols (359; 72%). Respondents reported not being blamed (341; 67%), subject to closer supervision (380; 76%) or punished (332; 67%).

**Table 3:** Outcomes of incident reporting

*Outcomes*	N	%
Blamed		
Not at all	341	68.61
Slightly	120	24.15
Missing/No response	36	7.24
Changed Protocol Guidelines		
Not at all	359	72.23
Slightly	95	19.11
Missing/No response	43	8.66
Changed Local level		
Not at all	373	75.05
Slightly	86	17.3
Missing/No response	38	7.65
Useful Feedback		
Not at all	426	85.71
Slightly	35	7.04
Missing/No response	36	7.25
Colleague Empathy		
Not at all	426	85.71
Slightly	36	7.24
Missing/No response	35	7.05
Closer Supervision		
Not at all	380	76.46
Slightly	77	15.49
Missing/No response	40	8.05
Punishment		
Not at all	332	66.8
Slightly	128	25.75
Missing/No response	37	7.45

Number of Observations: 497

The personal and professionals impacts of the event on respondents were apparent (see Table 4), with 386 (77%) reporting they had been affected professionally or personally in some way. A significant majority reported the event had impacted their mental health (416; 84%), substantial numbers of respondents said their physical health had also been affected (388; 78%). The professional implications of being involved in an event were demonstrated in impacts on job satisfaction (378; 76%) and their confidence in the ability (276; 56%).

**Table 4:** Personal Effect of involvement in Adverse Event

Covariates	N	%
Affected in general	386	77.67
Affected job satisfaction	378	76.06
Affected confidence in skills	268	53.92
Affected physical health	388	78.07
Affected mental health	416	83.70

Number of Observations (N): 497

As shown in Table 5, respondents commonly sought the support of peers at their own rank or level (338; 68%) and/or at a senior rank or level to them (266; 45%) as a result of their involvement in an event. Managers (219; 44%) and/or supervisors (98; 20%) were also commonly referred to for support, with family and friends (155; 31) also valued. Smaller groups of respondents sought support from a mentor (98; 20%) or referred to their own healthcare provider (108; 22%). Many respondents indicated they felt that their organisation offered adequate support to doctors and nurses involved in safety events (217; 43.66%). There was a significant correlation between those who did not perceive the support offered by the organisation was adequate and being involved in an event with serious patient harm (0.14, p- value 0.001).

**Table 5:** Sources of support *post* serious and minor adverse events/near misses

Variable	N
Peer	338
Senior Peer	266
Colleague from other professions	121
Friends and Family	155
Manager	219
Mentor	98
Patient	79
No one	32
Own Healthcare Provider	108

Number of Observations (N): 497

## Discussion

Our findings demonstrate significant personal and professional impact on doctors and nurses who are involved in AEs and near misses in the study hospital, which reflects international evidence [14, 15]. The pervasive impact of AE and error involvement was evident through the physical and mental health impacts, in addition to reducing job satisfaction. When compared to findings using the same survey instrument in the UK and in Australasia, the proportion of the Vietnamese sample reporting detrimental effects to confidence in their skills and mental health was similar to that reported in Australasia and in the UK. Detriment to physical health and job satisfaction however was higher than in studies in the UK and Australasia [24, 25]. A substantial group of respondents (34%) identified that they had not reported events that they should have, which compares with 25% of the UK and Australasian samples [24, 25].

Our data contribute to a growing body of work internationally, predominantly spanning the US, Canada and Europe, that has explored the ‘second victim’ phenomena of health professional impacts from involvement in safety events [26–29]. Data from our study sample in Viet Nam suggests that, unlike most developed country contexts, the majority of health professionals would talk to a manager and also to patients about safety events. These findings indicate that contextual factors relating to health system such as consumer empowerment, litigious culture, and organisational hierarchy, may influence actions that follow safety events [30]. In addition, as a trauma referral centre in high demand, health professionals in the study hospital have power in their relationship with patient, see large patient numbers and generally have less ongoing contact with them. Such factors may contribute to explaining the high proportion of disclosures made to patients.

The relevant absence of person-level, local-level or organisational-level actions resulting from a safety event emerged strongly in our data. When asked about experiences following a safety event, it was clear that, whilst respondents mostly did not report feeling blamed or punitive action, there was very little in the way of local or organisational improvement. The consistency of ‘not at all’ or ‘slightly’ responses to this group of items suggests that actions following a safety events are either rarely taken across the organisation or possibly that respondents did not wish to report these actions. A result of these data was that the degree to which organisational learning may occur as a result of a safety incident was unclear.

In keeping with the international evidence regarding clinician response to AE and error involvement, peers were identified as the most popular and valued source of support [31–33]. But unlike in the data emerging from developed countries, managers were equally identified as important sources of support. Conversely, discussions with patients and family members following an event were reported by less than half of the respondents in the present study, particularly in the context of serious events (35%) that would be associated with incident disclosure processes in many developed countries [34, 35]. Organisational support was identified as important in the context of AE and error involvement. Those who did not perceive the support offered by the organisation was adequate were significantly more likely to be those who were involved in an event with serious patient harm. As cross-sectional data, it is not possible to draw conclusive statements regarding the nature of this relationship but evidence in the research literature to date indicates two potential relationships between support and incidence of safety events. There is some evidence to suggest that those are better supported in their organisation, for example through mentorship, may be less likely to be involved in safety events or more serious events [13, 19, 36, 37]. Alternatively, being involved in an event with a harmful or more serious patient outcome may leave health professionals feeling unsupported by their organisation.

### Implications

The study findings have implications for organisational learning in relation to adverse events and errors, for supporting health professionals involved in these events, and for the development of effective incident reporting systems in Vietnamese hospitals. Optimal organisational learning is said to occur when an organisation looks beyond the events and circumstances immediately preceding an incident (single-loop learning) to explore the conditions within the organisation that have enabled the immediate circumstances to arise; characterised as double loop learning [38–40]. Limited evidence of any actions arising in response to an AE or near miss in the present study suggests that there is currently a lack of learning at a local or organisational level in response to the events occurring. An effective incident reporting system that promotes local and organisational learning activities after events is one mechanism to routinely embed learning as part of an incident management process. Systematic incident reporting and management can facilitate reflection on actions relating to the events arising but also to the latent factors that allowed the event to occur.

Establishing a strong patient safety culture within organisations at a national level is critical for the emergence of an effective incident management process. Fundamental to developing a strong patient safety culture, is the ability to identify and communicate safety concerns or challenges to the senior management of an organisation [13]. In order to progress work on patient safety cultures and incident reporting in Viet Nam, health professionals will need to be convinced not only that they will not be exposed to punitive action, but that learning and positive changes will occur as a result of reporting safety events. In progressing towards a strong safety culture, training of peers and managers (who are identified as the key sources of support following events) around impacts of AEs and errors may also contribute to culture change.

### Limitations

Whilst our findings reflect those of the other cross-sectional survey studies on this topic, a cross-sectional method is reliant on retrospective recall and may therefore lead to recollection bias. Cross-sectional self-reporting also limits the accuracy of information gathered regarding the severity and duration of emotions experienced in relation to an adverse event or near miss. However, the exploration of stable beliefs rather than specific experiences is of value in the context of the present study which seeks to know how experiences of involvement in AEs and errors has shaped reporting behavior and the emerging safety culture. The use of a responder sample may have influenced the resulting findings, despite the strong response rate; those who were strongly impacted or not impacted at all by their experiences may have declined to participate. The findings provide valuable insight into an issue that is difficult to discuss, particularly in developing country contexts such as Viet Nam. The management of adverse events and errors has significant implications for both clinician well-being and patient safety.

## Conclusion

Establishing a strong patient safety culture in Vietnamese hospitals is a necessary foundation for effective incident management, and subsequent learning to improve the safety of health care. The ability to identify and communicate safety concerns or challenges to the senior management of an organisation is essential. Work focusing on building a learning and just culture is required. As a first step health professionals need a mind-set where they believe that positive change to enhance safety will arise from talking openly about safety events. Healthcare leaders and managers have a critical role in affecting change in their organisations and sharing this knowledge within and between services.

## Data Availability

Study data is held by the study team and cannot be shared in accordance with ethical approval but may be sought from the lead author.

## Abbreviations

AE: Adverse event
UK: United Kingdom
US: United States of America
